# Eclampsia—Case, Cause, Treatment

**Published:** 1873

**Authors:** K. P. Moore

**Affiliations:** Knoxville, Ga.


### ECLAMPSIA—CASE, CAUSE, TREATMENT.

BY K. P. MOORE, M.D., KNOXVILLE, GA.

[Read Before the Middle Georgia Medical Society, July 16, 1873.]

May 9th, 1873, was called to Mrs. J. A. D., aged about twenty-seven, in primipara labor. Found her in first stage of labor, and apparently progressing well. Os about the size of a silver quarter, and dilatable. Health for some weeks unusually good. Recognized head presentation, but not position. Made myself easy, as I saw no cause for alarm. This was about five o'clock in the afternoon. Nothing unusual occurred until about midnight. Labor progressed slowly, and os gradually dilated—now about two and a half inches. I now recognized the presentation to be forehead. About midnight the pains began to cease, both in frequency and force. I thought nothing strange of it; went to bed, and knew nothing more of it until next morning. Went in on the morning of the 10th, before sunrise, and found my patient very stupid; some little writhing at a slight pain, but would go to sleep again as soon as pain was gone; could with difficulty arouse her long enough to answer, rather incoherently, questions asked—sleep becoming more and more profound, until about nine A.M., when she had a hard convulsion, followed by profound coma and stertorous breathing. I instantly bled her, and applied sinapisms and friction to extremities; in about two hours she became partially conscious, though greatly inclined to stupor. Gave large doses bromide of potassinm and chloroform. After some hours, with little or no pain, I thought it would be necessary to use obstetrical forceps. I sent for my friend, Dr. J. E. Cook, to bring them.

By three o'clock P.M. pains a little increased, and os fully dilaled; debated the propriety of giving ergot, but as I was looking every minute for Dr. Cook, decided to wait for him. Dr. Cook arrived by four P.M. Pains slightly increasing, and child slowly advancing. Decided to give ergot, and wait on nature; uterus responded but little to the ergot, though several drachms of the fluid extract were given. Two hours later the patient became very nervous, and probably had a light convulsion. Decided to proceed at once to deliver. Dr. Cook succeeded in passing the fingers of both hands around the head of the child, and delivered it still-born.

It was now discovered that there were twins. A short time was allowed, and no pains supervening, Dr. Cook, at my request, very skillfully delivered the second child by forceps—also still-born. Patient all the while entirely unconscious; she appeared at this time quite prostrated. Gave a little toddy, and circulation soon rallied, and she seemed to be doing well; a little chicken tea was given, and patient left to herself.

Next morning, the 11th, she was in semi-conscious condition; took a little chicken tea, and was thought to be doing well. Dr. Cook left me in good hopes. Gave an occasional dose bromide of potassium. By twelve M. she became comatosed, and continued to grow worse until four P.M., when death forever released her from her pains. There seemed to be perfect inertia on the part of the uterus from an overload. Children weighed about eight pounds each. There were never any expulsive pains.

On the morning of the 10th, about an hour or more from the first convulsion, I drew off about six ounces of urine, and subjected it to test for albumen by heat and carbolic acid. The carbolic acid threw down a perfect cloud of coagulated albumen. The specimen by heat I now present to the Society, which is, as you see, nearly all coagulated; out of about an ounce of urine submitted to heat, not more than half a drachm remained in fluid state. My friend, Dr. Cook, agreed with me that the like had never before been seen by our eyes. About fifteen hours after delivery I again drew off some urine, and submitted it to the carbolic acid test; and while albumen was in abundance, yet I was forcibly impressed with the fact that there was perhaps not more than half as much as before. Regret that I was not more careful in my second day's test, and had not submitted *it* also to heat, and preserved the specimen.

Never did I give up a patient with as much reluctance, and it naturally aroused in my mind an inquiry as to the cause, pathology and treatment of this fearful disease. So far as I have been able to investigate the subject, I find no theory or explanation, as to cause, to which I can cling, other than that upon which we all agree—that the convulsions depend upon brain-poisoning, the result of overcharging the blood with albumen; but as to the source from whence this albumen comes, I present to the Society, for their consideration, a theory entirely new, so far as I know, and original with me, and which, too, would very much modify my treatment of a case prior to confinement. Of course, predisposing and exciting causes are not to be overlooked. It has been very justly remarked by Dr. Locock, "That the immediate or exciting causes of puerperal convulsions are very obscure."

Among the predisposing causes might be mentioned epilepsy, hysteria, or any congenital or other predisposition to convulsion in early life. Some of the exciting causes are atmospheric influences; sudden mental emotions, either of fright or joy; an overloaded stomach, or, if in labor, a too long-continued hard pain. Dr. Marshall Hall maintains that, "So long as the spinal system is not involved, no injury of the cerebrum or cerebellum can cause convulsions. "Puerperal convulsions," says he, "depend for its cause upon morbid irritation of the true spinal system, more especially of the medulla oblongata, propagated to it from mucous surfaces through the incident nerves of the excito-motor system.

Dr. Murphy holds that "Puerperal convulsions is a beautiful illustration of the reflex mucous function, excited by morbid irritation of the uterus."

Says Dr. Tyler Smith: "True puerperal convulsions can only occur when the central organ of the system, the spinal marrow, has been acted on by an excited condition of an important class of its incident nerves—viz., those passing from the uterine organs to the spinal center; such excitement dependent on pregnancy, labor, or the puerperal state."

Hamilton and Demauret, without probably appreciating the source of the trouble, say that "puerperal convulsions were liable to be preceded by anasarca."

Drs. Simpson and Lever first undertook to connect this dropsy to that condition of the kidney which gives rise to albumen, This theory has also been accepted by Colier and Bouchut, Roger, Depaul, Cazeaux, and indeed nearly all the more recent investigators.

A correspondence with some of the prominent teachers of obstetrics and gynæcology in our country, besides conversations with many physicians with whom I have come in contact since I have had this subject under study, acquaints me with the fact that the profession is now leaning strongly to the uræmic theory. Now, while I fully adopt the opinions of Simpson, Roger, Cazeaux and others, as to the fact that the presence of albumen in the blood is the real cause of eclampsia, I differ with them widely as to the source from whence this albumen comes; now if this albumen is dependent upon a diseased kidney, as held by the authors recited, why is it not at once eliminated with the urine, as is usually the case with albuminuria? How does it enter the blood, especially in such abundance as we sometimes find it? If the amount of albumen which we sometimes find present in the blood of puerperal women could be dependent upon a pathological kidney, certainly we would have more constitutional disturbance than we usually find present, and instead of the usual plumpness, fine fat appearance and extraordinary good health which is sometimes present, and which was present in my patient, we would have the peculiar distressed appearance, and fast failing constitution of a patient suffering with morbus Brightii.

One other reason for excluding this theory of a diseased kidney. We would scarcely expect a patient to be so soon relieved of so terrible a malady in so short a time as we find a puerperal woman recovering after confinement.

Having, we think, fully excluded the theory of organic renal lesion, I next notice the opinions of Dr. Cormick and those who hold to the opinion that the albumen is the toxicological result of non-elimination on the part of the kidneys, from congestion, caused by mechanical pressure. I exclude this theory upon the ground that instead of there being less urine and excretions passed, the urinary organs are really over-taxed, and a great deal more, than in an unimpregnated state, is being passed.

Dr. Cormick and his disciples hold to the opinion that this congestion disqualifies the kidneys for the performance of their function, and a failure to perform this function, allows this abnormal element, which otherwise would be eliminated, to accumulate in the blood to the extent of producing brain-poisoning. I cannot see how they can be such hard masters on the poor over-worked kidneys, and accuse them of not doing their duty, when an examination of the quantity and quality of the urine will convince us at once that they are making herculean efforts to free the blood of the terrible elements which seem to threaten the very life of the patient.

Excluding all these theories, we come now to look for an explanation of this accumulated albumen in another direction, which direction I am somewhat surprised to find has not attracted the notice of the profession before. I am persuaded that a careful glance at the physiological developments of the fœtus will throw much light upon the subject. Says Dalton:

"In early fœtal life, the fœtus is inclosed between two distinct membranes, the ammion and chorion, the umbilical vesicle being between the two, and the rest of the space between the ammion and chorion is occupied by a semi-fluid gelatinous material, somewhat similar in appearance to the vitreous body of the eye," from which the umbilical vesicle draws nourishment. The chorion now becomes shaggy, with numerous villosities. These villi increase the extent of the surface of the chorion, and assist in the absorption of fluids from without. These villosities become permeated by blood vessels, and after being thickly studded on the surface of the chorion, are afterwards themselves taken up, (probably by absorption) for two-thirds the whole extent of the chorion, the remaining third becoming augmented in size is largely concerned in the formation of the placenta. I wish it to be remembered that the fluids absorbed by these villi, and afterwards the absorbed villi themselves, are of an albuminous character, and that they enter into the fœtal circulation.

It is also a physiological fact that the decidua vera, which early in fœtal life, is very large and thick, afterwards becomes absorbed, and only the decidua reflexa continues to grow and assist in the formation of the placenta. Now, as we have seen, the fœtus, during development, depends for nourishment upon this gelatinous material and upon the lining membrane of the maternal uterus, all of which is highly albuminous.

We further learn from Dalton that, "After this the ammion enlarges faster than the chorion and encroaches upon this layer of gelatinous material, at the same time an *albuminous* fluid, the amnistic fluid, is exuded into its cavity in constantly increasing quantiiy, so as to accommodate the movements of the fœtus."

As the placenta becomes more developed, and the fœtus receives more of its support by endosmose from the maternal circulation, it also eliminates, by exosmose, its excrementitious matters through the maternal blood. Now if, as we have already seen, a large amount of albuminous material has already entered the fœtal circulation, also that the fœtus floats in a perfect flood of albuminous fluid, is it not very reasonable to suppose that the albumen which we find so abundant in the puerperal state comes from the fœtus, and not from the fault of a lazy, indifferent set of kidneys?

Looking to this source, then, for an explanation of eclampsia, our treatment of course would be greatly modified, for we would not give medicines for morbus Brightii when no such condition existed. What, then, are the indications for treatment? Remove it from the blood, of course, is the indication. How is this to be done? I cannot see any other plan than by blood-letting, thereby removing the vitiated blood and endeavoring to supply its place by tonics and good nourishments; I would then bleed occasionally, and otherwise adopt the supportive treatment.

Of course, predisposing and exciting causes are not to be overlooked and guarded against. The bowels should be kept open; heavy meals, *especially of indigestible*, substances should be strictly guarded against. Mental excitement of any character should be avoided; proper exercise in open air, and such other hygenic measures, adopted as will suggest themselves to the minds of all intelligent physicians. I cannot close without returning thanks to Dr. Cook, who I have often found to be all I could ask in a professional brother.

